# Winter Railroading

**Published:** 1876-04

**Authors:** 


### WINTER RAILROADING.

SUCH multitudes travel in rail cars in winter time, it will be a public benefit to make some statements in its bearing on health. To regulate the temperature of any car to suit the hundred different persons who occupy it, is simply impossible. Only general principles can be profitable and practical.

It is better that the car should be too warm than too cold, for the many who come into it in a more or less heated condition, from various causes, too well known to be enumerated. A person terminating exercise in a very warm room cannot take cold. A person terminating exercise causing the slightest moisture on the surface, will always take cold within fifteen, often within five, minutes after sitting still in a cold apartment; and if continued, an attack of pleurisy, or inflammation, or congestion of the lungs, is an almost certain event, from either of which results a life-long inconvenience, if not, indeed, a speedy death. Therefore, as to all persons entering a car at the beginning of a journey, it is safer beyond comparison, that it should be too warm than too cold.

Persons sitting in a cold car for a time sufficient to allow them to get thoroughly chilled, will scarcely fail to suffer from an attack of some acute disease, in spite of a subsequent warming up by exercise or otherwise, while it is well known that persons may remain for hours in an apartment heated to a hundred degrees and over, without any permanent discomfort, if they are careful to cool off slowly.

But as the cars may be very hot in midwinter, and passengers are put down at every station, and often without any fire to go to, it is most of all important to know how to conduct oneself without injury under the circumstances. It is only necessary to have all the clothing adjusted—hat, gloves, everything—before the cars stop; as soon as they stop, shut your mouth, open the door, and run as fast as you can to your destination, or the first available house, keeping the mouth remolutely shut, if possible, until you get within doors, and then remain with all your clothing on for ten or fifteen minutes.

The running keeps the blood warm, and to the surface.

The closing of the mouth sends the cold air by the circuit of the nose, and heats it before it reaches the lungs.

The retention of the clothing allows the circulation to become natural slowly, and while so, no one can take cold.

With these precautions, the more a person travels by railroad the more hearty will he become, and, eventually, will not take cold in a year's travel.

In winter railroading the feet require most attention. The floor of the car is the coldest part of it, under any circumstances, while a single plank separates them from a zero temperature, it may be. Persons will greatly consult their comfort by keeping their feet on the foot-boards, and in addition, have the feet and legs well wrapped in a substantial blanket or other covering. It is vastly better to shawl the feet than the shoulders in a rail car.

